# C19orf66 interrupts Zika virus replication by inducing lysosomal degradation of viral NS3

**DOI:** 10.1371/journal.pntd.0008083

**Published:** 2020-03-09

**Authors:** Yun Wu, Xinyu Yang, Zhicheng Yao, Xinhuai Dong, Danrui Zhang, Yiwen Hu, Shihao Zhang, Jiajie Lin, Jiahui Chen, Shu An, Hengming Ye, Shuqing Zhang, Ziying Qiu, Zhenjian He, Mingxing Huang, Guohong Wei, Xun Zhu

**Affiliations:** 1 Key Laboratory of Tropical Disease Control (Sun Yat-sen University), Ministry of Education, Guangzhou, China; 2 Department of Microbiology, Zhongshan School of Medicine, Sun Yat-sen University, Guangzhou, China; 3 Depatment of Haematology, The Third Xiangya Hospital, Central South University, Changsha, China; 4 Department of General Surgery, The Third Affiliated Hospital of Sun Yat-sen University, Guangzhou, China; 5 Department of Center for Translational Medicine, Shunde Hospital, Southern Medical University, Foshan, China; 6 Department of Basic Medicine, Zhongshan School of Medicine, Sun Yat-sen University, Guangzhou, China; 7 Changsha Customs District P.R. China, Changsha, China; 8 Department of Clinical Medicine, Zhongshan School of Medicine, Sun Yat-sen University, Guangzhou, China; 9 School of Public Health, Sun Yat-sen University, Guangzhou, China; 10 Department of Infectious Diseases, The Fifth Affiliated Hospital of Sun Yat-sen University, Zhuhai, China; 11 Department of Endocrinology, The First Affiliated Hospital of Sun Yat-sen University, Guangzhou, China; University of Texas Medical Branch, UNITED STATES

## Abstract

The rapidly emerging human health crisis associated with the Zika virus (ZIKV) epidemic and its link to severe complications highlights the growing need to identify the mechanisms by which ZIKV accesses hosts. Interferon response protects host cells against viral infection, while the cellular factors that mediate this defense are the products of interferon-stimulated genes (ISGs). Although hundreds of ISGs have been identified, only a few have been characterized for their antiviral potential, target specificity and mechanisms of action. In this work, we focused our investigation on the possible antiviral effect of a novel ISG, C19orf66 in response to ZIKV infection and the associated mechanisms. We found that ZIKV infection could induce C19orf66 expression in ZIKV-permissive cells, and such an overexpression of C19orf66 remarkably suppressed ZIKV replication. Conversely, the depletion of C19orf66 led to a significant increase in viral replication. Furthermore, C19orf66 was found to interact and co-localize with ZIKV nonstructural protein 3 (NS3), thus inducing NS3 degradation via a lysosome-dependent pathway. Taken together, this study identified C19orf66 as a novel ISG that exerts antiviral effects against ZIKV by specifically degrading a viral nonstructural protein. These findings uncovered an intriguing mechanism of C19orf66 that targeting NS3 protein of ZIKV, providing clues for understanding the actions of innate immunity, and affording the possible availability of new drug targets that can be used for therapeutic intervention.

## Author summary

ZIKV represents a serious threat to global health with particular relevance to microcephaly and other congenital abnormalities in newborns, and Guillain-Barré syndrome, meningoencephalitis, multi-organ failure in adults. Despite the global health threat of Zika virus infection, there is currently no vaccine or effective antiviral therapy available for the disease. As widely recognized, interferon signaling is key to establishing a strong antiviral state in host cells, mainly mediated through the anti-viral effects of numerous interferon-stimulated genes (ISGs). This work described our novel finding of the antiviral effect of a novel ISG, C19orf66, and its underlying mechanisms. We identified C19orf66 as a novel ISG that exerts antiviral effects against ZIKV by specifically interacting and colocalizing with the ZIKV nonstructural (NS) protein NS3, which inducing NS3 degradation via a lysosome-dependent pathway. Thus, this work broadens the understanding of the pivotal roles of C19orf66 in the interaction between the host and ZIKV, which might further provide a rational basis for developing novel anti-ZIKV strategies.

## Introduction

Zika virus (ZIKV) belongs to the family *Flaviviridae* (genus *Flavivirus*), which is mainly transmitted by *Aedes* mosquitoes. In the last decades, mosquito-borne flaviviruses, including dengue virus (DENV), West Nile virus (WNV), and, more recently, ZIKV have caused significant epidemic outbreaks [1]. In late 2013 or early 2014, the largest Zika pandemic to date began in the Americas, in particular Brazil, and by August 2018, more than 84 countries and territories had reported cases of ZIKV transmission [2]. Despite that the major clinical syndromes caused by ZIKV are self-limited acute fever, rash, arthralgia, and conjunctivitis, until now ZIKV represents a serious threat to global health and was declared an international emergency by the World Health Organization, with particular relevance to microcephaly and other congenital abnormalities in newborns, and Guillain-Barré syndrome, meningoencephalitis, and multi-organ failure in adults, which is the most striking and has never been observed in infection with any other flavivirus, like DENV, or yellow fever virus (YFV) [1, 3]. However, there are no clinically approved vaccines or drugs available for ZIKV infection or its associated diseases, and patients are dependent on innate and adaptive parts of the host immune response to fight the infection [1]. Hence, there is a pressing need for a comprehensive understanding of the molecular pathogenesis of ZIKV and the host immune response, to aid in the development of effective vaccines and antiviral therapies.

The genome of ZIKV is a positive-sense and single-stranded RNA, which encodes a single viral polyprotein. Then the polyprotein is processed by both viral and cellular proteases to produce three structural proteins including capsid (C), pre-membrane (prM, the precursor form of M), and envelope (E), and seven nonstructural (NS) proteins (NS1, NS2A, NS2B, NS3, NS4A, NS4B, and NS5) [1]. While the structural proteins and genomic RNA form the virion, the nonstructural proteins are necessary for viral RNA replication, virion assembly and subverting the antiviral innate immune response of the host to establish infection [1, 4]. Among the nonstructural proteins, NS3 junctions with the cofactor NS2B to play a vital role in these processes, which consists of an N-terminal protease domain and a C-terminal helicase domain. The N-terminal protease domain of NS2B/3 is responsible for the proteolytic cleavage of the viral polyprotein and the C-terminal helicase domain supports the separation of the daughter RNA from the template strands and unwinds the RNA secondary structure in the 3’non-translated region (NTR) during viral replication [4]. More importantly, several ZIKV-encoded proteins likely play an important role in the emergence and pathogenesis of ZIKV in humans [3]. It has been characterized that mutations in the prM [5] and NS1 [6] proteins have enabled ZIKV to become more virulent and transmissible. Mounting evidence has supported that innate immunity is thought to govern both ZIKV replication and pathogenesis. It has also been reported that the interaction of ZIKV NS3 protease with NS2B mediates the cleavage of STING to prevent the production of type I IFN, subvert the host innate antiviral response and promote viral replication [7].

Type I interferons (IFNs-I) are cytokines of the innate immune system that have been recognized as the first line of defense against viral infection [8]. Rapid and robust induction of IFN-I is a critical event in the host antiviral innate immune response [8]. IFNs-I themselves possess no direct antiviral activity, instead they acts in a paracrine and autocrine fashion to bind the heterodimeric transmembrane receptor termed the IFN-α/β receptor (IFNAR), which then activates more than 300 interferon-stimulated genes (ISGs) via the JAK-STAT signaling pathway [8]. ISGs have been reported to encode proteins with potential direct antiviral activity through multiple mechanisms, including the inhibition of viral transcription, translation and replication, the degradation of viral nucleic acids and the alteration of cellular lipid metabolism [8]. However, many ISGs are still undiscovered, and their antiviral mechanisms need to be further investigated and thoroughly characterized. Recent studies have indicated that several ISGs, such as IFITM1 [9], IFITM3 [9], PARP12[10] and Viperin [11] could inhibit ZIKV replication. A recent high-throughput cDNA library derived from type I IFN-treated human cells screen was used to identify a set of genes that form a novel regulatory network of host factors that mediates antiviral effects [12, 13]. Among them, a factor called C19orf66 (also known as IRAV, UPF0515, RyDEN) was suggested to be a novel ISG, the products of which can suppress DENV, WNV, hepatitis C virus (HCV) and Chikungunya virus (CHIKV) replication *in vitro* [12, 13]. Suzuki Y *et al* [12] reported that C19orf66 interfered with the translation of DENV via binding to viral RNA, positive modulators PABPC1 and LARP1, leading to the inhibition of viral replication in infected cells. Balinsky *et al* [13] found that C19orf66 co-localized with the DENV replication complex and restricted DENV through influencing the processing of viral RNA. Whether C19orf66 participates in the host innate immune response to defend against ZIKV infection is still unknown and the underlying mechanism is deserved to be investigated.

Lysosomes are membrane-bound organelles that are found in the cytoplasm of most cells and function as the "stomachs" of these cells [14]. They contain various hydrolytic enzymes that break down a wide variety of extra- and intra-cellular materials including proteins, nucleic acids, lipids, and carbohydrates [14]. Both extracellular materials brought into the cell by endocytosis, autophagocytosis, or phagocytosis, and obsolete intracellular materials including macromolecules or infectious pathogens are degraded in the lysosome, which are essential for innate immunity recognition, antigen presentation, and pathogen elimination [14]. In recent years, substantial links between virus pathogenesis and lysosomal biology have been reported, including influenza virus [15], Semliki Forest virus [16], adenovirus [17], and hepatitis A virus [18]. These viruses can cause lysosomal rupture and releasing hydrolase enzymes into the cytosol, which increase the cell death rate, and enhance viral replication in the infected host cells. Nonetheless, there were evidences indicate that viruses can escape innate immune system by lysosomal degradation of the key signal molecules. Rui Zhang et.al [19] reported that pseudorabies virus could utilize its encoded dUTPase UL50 to induce IFNAR1 degradation and inhibit type I IFN signaling in an enzymatic activity-independent manner. Sebastian Aguirre et. al [20] found that the DENV NS2B protease cofactor targets the DNA sensor cyclic GMP-AMP synthase (cGAS) for lysosomal degradation to avoid detection by mitochondrial DNA during infection.

In this study, we identified C19orf66 as a novel ISG that could be induced by ZIKV infection and IFN-I treatment. This work demonstrated that the overexpression of C19orf66 could suppress ZIKV replication in host cells, on the contrary, cells depleted C19orf66 exhibited an increase of viral replication. Further investigation revealed that C19orf66 could interact with the NS3 protease domain and mediated ZIKV NS3 degradation via a lysosome-dependent pathway. Collectively, our data suggested that C19orf66 might be a novel ISG and plays a critical role in suppressing ZIKV infection by specifically degrading a viral nonstructural protein.

## Materials and methods

### Ethics statement

All animal studies were approved by the SYSU Institutional Animal Care and Use Committee (SYSU IACUC) under the number NO. 2017–118, and all animal experiments were performed in accordance with the guidelines of Animal Research: Reporting of In Vivo Experiments (ARRIVE) and guidelines approved by SYSU IACUC, and conducted in a biological safety protection laboratory.

### Plasmids and transfection

Plasmids encoding human C19orf66 with a Myc epitope tag, ZIKV NS3 with a Flag epitope tag, DENV NS3 with an HA epitope tag, ZIKV NS2B/3 with a His epitope tag, STING with an HA epitope tag, and ubiquitin with an HA epitope tag, were amplified by reverse transcription-PCR (RT-PCR) and cloned into the pCDEF vector. Truncated forms of NS3 (amino acids [aa] 1 to 167, and 168 to 617) with Flag epitope tags were amplified from the full-length template and cloned into the pCDEF vector. All constructs were verified by DNA sequencing. The PCR primers used in this study are summarized in S1 Table in the supplemental material. The small interfering RNAs (siRNAs) against C19orf66, the targeting sequences of which are provided in S1 Table were purchased from RiBoBio (RiBoBio Inc, Guangzhou, China). Transfection of the siRNAs and plasmids at indicated concentrations were performed using the Lipofectamine 3000 reagent (Invitrogen, Carlsbad, CA) following the manufacturer’s instructions. To assess the expression of C19orf66 protein, cell lysates were harvested 48 h after transfection and determined by Western blotting with corresponding specific antibodies. Inhibitors including MG-132, chloroquine, NH_4_Cl, and 3-MA were purchased from Sigma-Aldrich (Sigma-Aldrich, St. Louis, MO).

### Cell culture and virus

hNPC (human neural progenitor cell) was purchased from Merck (NJ, USA), and hNPC cells were cultured according to the manufacturer's instructions. SNB-19 (human glioblastoma cell line), A549 and 293FT cells were obtained from the Cell Bank of the Chinese Academy of Sciences (Shanghai, China). Cells were cultured at 37°C with 5% CO_2_ in Dulbecco’s modified Eagle’s medium (DMEM) (Invitrogen, Carlsbad, CA) supplemented with 10% fetal bovine serum (FBS) (Gibco, Carlsbad, CA), 2 mM L-glutamine, 100 μg/ml streptomycin, and 100 units/ml penicillin (Invitrogen, Carlsbad, CA). *Aedes albopictus* C6/36 cells (ATCC CRL-1660) were maintained at 28°C with 5% CO_2_ in DMEM supplemented with 10% FBS. Zika virus strain ZG-01 (GenBank accession number KY379148.1) was isolated by our group in 2016 [21, 22], and was propagated in C6/36 cells. Viral stocks were stored at -80°C and titrated on C6/36 cells.

### ZIKV titration and plaque assay

Zika virus titers were determined by a plaque assay performed on Vero cells by following the procedures as described previously [10]. Briefly, Vero cells were seeded in 12 well plates and used for infection when the cells were grown to 90% confluence. Following washing with phosphate-buffered saline (PBS), confluent monolayer cells were absorbed by virus stocks or samples that were serially diluted for 1 h at 37°C with 5% CO_2_. After the virus inoculum was removed and cells were washed with PBS, followed by overlaid with 1% methyl cellulose. Cells were further incubated for 5 days, after which they were fixed in 4% paraformaldehyde, followed by stained with 1% crystal violet in 20% ethanol for plaque visualization. Visible plaques were counted and the virus titers were expressed as plaque forming units (PFU) per milliliter.

### *In vivo* murine infections

Type-I interferon receptor-deficient (*Ifnar1*^-/-^) mice that succumbed to ZIKV infection were used as a useful ZIKV mouse model, and all mice were bred in pathogen-free animal facilities at Sun Yat-Sen University (SYSU). Mice that were approximately 5 weeks of age were used for these experiments. According to a previously described method [22, 23], *Ifnar1*^*−/−*^ mice were injected intraperitoneally with 1×10^5^ plaque-forming units (PFUs) of ZIKV in 100 μl, which were monitored daily for morbidity and mortality.

### Real-time RT-PCR

Total cellular RNA was prepared with TRIzol (Invitrogen, Carlsbad, CA) according to the manufacturer’s suggested protocols. For the first-strand cDNA synthesis, 500 ng of total RNA was reverse transcribed using random hexamer primers. Real-time RT-PCR was carried out by using FastStart Universal SYBR Green Master Mix or Roche LightCycler480 Probes Master (Roche, Basel, Switzerland). All readings were normalized to the level of glyceraldehyde-3-phosphate dehydrogenase (*GAPDH*) mRNA. The primer sets used for real-time RT-PCR are shown in S1 Table in the Supporting Information.

### Western blot analysis

Cells were lysed with sampling buffer (50 mM Tris-HCl [pH 7.4], 1 mM PMSF, 10% glycerol, 6% SDS, 5% beta mercaptoethanol and 0.1% bromophenol blue), and the protein concentrations were measured with the BCA protein assay (Thermo Fisher Scientific, Rockford, IL). Protein samples were separated by sodium dodecyl sulfate polyacrylamide gel electrophoresis (SDS-PAGE) and transferred onto a PVDF membrane. Nonspecific antibody binding sites were blocked with 5% non-fat milk in Tris-buffered saline (TBS) (20 mM Tris-HCl, pH 7.6, 135 mM NaCl, and 0.1% Tween-20) for 1 h at room temperature, and then the membranes were incubated with the following primary antibodies: anti-E (GeneTex, Alton Pkwy Irvine, CA), anti-NS3 (GeneTex, Alton Pkwy Irvine, CA), anti-NS2B (GeneTex, Alton Pkwy Irvine, CA), anti-C19orf66 (Abcam, Cambridge, UK), anti-Myc (Proteintech, Rosemont, IL), anti-Flag (Sigma-Aldrich, St. Louis, MO), anti-HA antibody (Sigma-Aldrich, St. Louis, MO), anti-LAMP1 (Abcam, Cambridge, UK), anti-LC3B(Abcam, Cambridge, UK), anti-His (Abcam, Cambridge, UK), anti-P65(Abcam, Cambridge, UK), anti-β-actin (Abcam, Cambridge, UK), and anti GAPDH (Abcam, Cambridge, UK). The membranes were incubated with horseradish peroxidase-conjugated secondary antibody, and signals were detected by enhanced chemiluminescence using a commercial kit (Thermo Fisher Scientific, Rockford, IL) by following the manufacturer's instructions. The intensities of the bands of interest on the PVDF membranes were quantitatively calculated with Quantity One 4.6.3 measurement software (Bio-Rad, Hercules, CA).

### Immunofluorescence assay

Cells were plated onto coverslips in a 24-well plate and transfected with the indicated plasmids (NS3-Flag, 1000 ng, C19orf66-Myc, 250 ng). At 48 h post-transfection, cells were washed once with phosphate-buffered saline (PBS) and fixed in 4% paraformaldehyde in PBS. Cells were permeabilized with 0.2% Triton X-100 and blocked for 30 min at room temperature with 10% bovine serum albumin (BSA) in PBS, followed by incubation with the primary antibody for 2 h. After three washes with PBS containing 0.1% Tween 20 (PBST), cells were incubated with rhodamine-conjugated secondary antibodies (Jackson ImmunoResearch Laboratories, West Grove, PA) or with Alexa Flour 488-conjugated secondary antibodies (Life Technologies, Grand Island, NY) for 30 min and then incubated with 4-,6-diamidino-2-phenylindole (DAPI) for 5 min. Finally, the coverslips were washed extensively and fixed onto slides. Before the indicated time point, the cells were stained with LysoTracker (L12492, LIFE) for 2 hours. Images were taken under a Zeiss Axio Imager Z2 and a LSM780 confocal microscope (Carl Zeiss MicroImaging GmbH, Jena, Germany).

### Coimmunoprecipitation assay

Cells were transfected with the indicated plasmids (1 μg/ml) by using a standard calcium phosphate coprecipitation method for the indicated times. Then, cells were lysed with protein lysis buffer containing 25 mM HEPES, 150 mM NaCl, 1 mM EDTA, 2% glycerol, 1% NP-40, and a cocktail of protease and phosphatase inhibitors (Roche, Basel, Switzerland), with or without RNase A (Genstar, San Francisco, CA). The lysates were incubated with the indicated antibodies or IgG antibody overnight at 4°C. Subsequently, the precipitates were washed five times with wash buffer containing 20 mM HEPES, 150 mM NaCl, 1 mM EDTA, 1 mM EGTA, 2% glycerol, and 0.1% NP-40; resuspended in sampling buffer; and examined by Western blotting.

### Lysosome enrichment and acid phosphatase activity assay

Lysosome enriched fractions were isolated from cultured cells using the Lysosome Enrichment Kit for Tissue and Cultured Cells (Thermo Fisher, Waltham, MA) according to the manufacturer’s protocol. Briefly, the cells were harvested and were mixed with 800 μl of Lysosome Enrichment Reagent. Then cell suspension was sonicated on ice, with a sufficient number of bursts being applied for effective cell lysis. Following the addition of 800 μl of Lysosome Enrichment Reagent B, centrifugation was performed and supernatant was collected in a new tube. In an ultracentrifuge tube, the OptiPrep gradients were prepared in descending concentrations: 30%, 27%, 23%, 20% and 17%. The sample was overlaid with containing the 15% OptiPrep Media on top of the density gradients. After ultracentrifuging the samples at 145,000 × g for 30 mins, the lysosome pellet was collected by adding 1 ml of the Gradient Dilution Buffer. The activity of acid phosphatase activity was assayed by a commercially available kit (Sigma-Aldrich, St. Louis, MO) according to the manufacturer's instructions. Briefly, the lysosomes were incubated with para-nitrophenyl phosphate at 37°C for 10 min after enrichment, then the stop solution was added to stop the process. Samples were measured with a microplate reader at an absorbance of 405 nm. The purity of the lysosomes was also assessed by Western blotting using antibodies against LAMP1 (a lysosomal marker).

### Statistical analysis

All statistical analyses were performed using the SPSS 20.0 statistical software package. The data accorded with normal distribution and homogeneity of variance provided in the text were presented as the means ± standard deviations (SDs) derived from three independent experiments. Comparisons between two groups were evaluated by a two-tailed Student’s t test. For pairwise multiple comparisons, statistical analyses were performed on triplicate experiments using one-way ANOVA followed by Dunnett’s multiple comparison test. Non-normal distributed data with uneven variance were expressed as the means and 95% confidence intervals (CIs). Mann-Whitney U test was applied for multiple comparisons. *p* values < 0.05 were considered statistically significant.

## Results

### C19orf66 expression is upregulated by ZIKV infection

**Fig 1:**
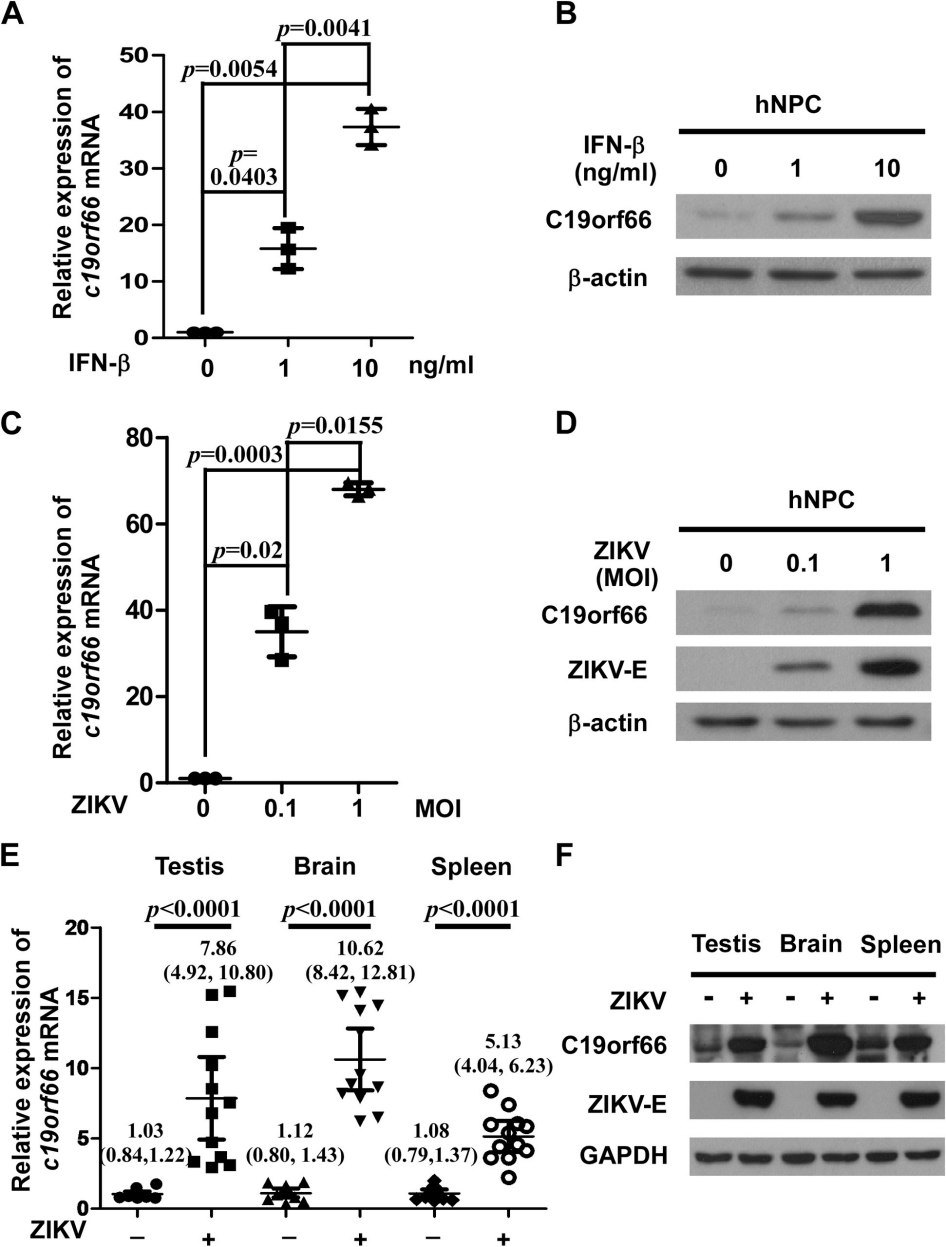
ZIKV infection or IFN-β treatment induces C19orf66 expression. hNPC cells were stimulated with IFN-β for 24 hours (h) at a concentration of 0, 1, or 10 ng/ml. And hNPC cells were infected with ZIKV at an MOI of 0.1, or 1 or without ZIKV. The mRNA and protein expressions of C19orf66 were detected by real time RT-PCR, and normalized to the expression of GAPDH in each sample (**A, C**) and Western blotting (**B, D**) respectively. The data shown in Fig 1A and 1C were expressed as the means ± SDs from three repeat experiments, and were analyzed by one-way ANOVA followed by Dunnett’s multiple comparison test. *Ifnar1*^-/-^ mice were challenged with Zika virus (1x10^5^ PFUs) via intraperitoneal injection for 6 days. The mRNA and protein expression levels of C19orf66 in the mouse testis, brain and spleen were detected by real time RT-PCR (**E**) and Western blotting (**F**). The data are representative of three independent experiments as seen in Fig 1B, 1D and 1F. The results shown in Fig 1E were expressed as the means and 95% confidence intervals (CIs), and the Mann-Whitney U test was applied for comparisons.

As C19orf66 had been proven to be an interferon-stimulated gene (ISG) [12, 13], we examined the expression of C19orf66 in hNPC after IFN-β treatments. The mRNA and protein levels of C19orf66 were upregulated by treatment with IFN-β at concentrations of 1 ng/ml or 10 ng/ml (Fig 1A and 1B), as indicated by 15.81±3.62 or 37.33±3.20 fold increases respectively compared with those in the control group. To investigate the role of C19orf66 in the context of actual ZIKV infection, hNPC cells were infected with ZIKV, and the cell lysates were analyzed for C19orf66 expression by real-time RT-PCR and Western blotting. The results showed that ZIKV infection of hNPC at an MOI (0.1 or 1) led to transcriptional and translational up-regulation of C19orf66 by 34.99±5.80 or 68.01±1.49 fold respectively compared with the levels in the control group (Fig 1C and 1D). Furthermore, to study the interplay between ZIKV and the host immune system, we adopted type-I interferon receptor-deficient (*Ifnar1*^-/-^) mice to generate a ZIKV infection model *in vivo*. We measured C19orf66 production (at both the protein and mRNA levels) in testis, brain and spleen tissues from mice infected with ZIKV or mock at 6 days post-infection. As shown in Fig 1E and 1F, the relative mRNA-level and protein-level expression of C19orf66 increased remarkedly in the testis, brain, and spleen from ZIKV-infected mice. Taken together, these results suggested that C19orf66 as an ISG, up-regulated by ZIKV infection.

### C19orf66 suppresses ZIKV replication

**Fig 2:**
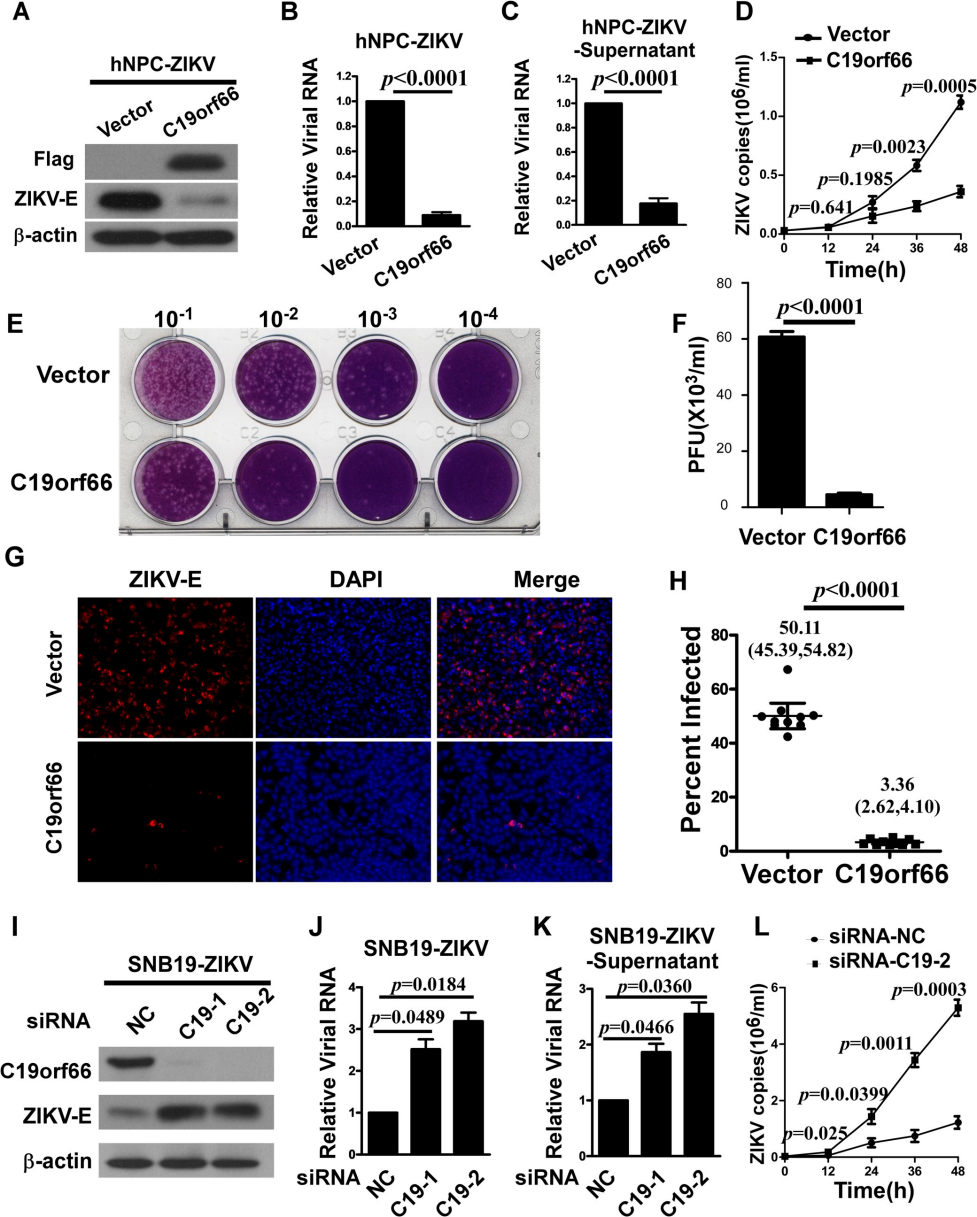
C19orf66 restricts ZIKV infection in cells. **(A)** hNPC cells were stably transduced with a retrovirus vector expressing C19orf66 or a vector control, infected with ZIKV at an MOI of 1, and then collected at 48 hours post infection. C19orf66 overexpression, the viral envelope protein of ZIKV and β-actin protein were verified by Western blotting analysis. The indicated cellular viral RNA **(B)** and supernatant viral RNA **(C)** levels were determined by using real time RT-PCR. The expression levels were normalized to the level of GAPDH. (**D**) C19orf66-overexpressing or control vector-transfected hNPC cells was infected with ZIKV at an MOI of 1, and then were collected at the indicated times. The number of cellular Zika virus RNA copies was determined by using quantitative real time RT-PCR. (**E, F**) The role of C19orf66 proteins in ZIKV infection was evaluated by using a plaque-forming assay. The results shown in Fig 2B, 2C and 2F were expressed as the means ± SDs from three repeat experiments, and comparisons were made by a two-tailed Student’s t test. **(G)** C19orf66-overexpressing hNPC cells and control cells were infected with ZIKV, and then stained with an anti- ZIKV E antibody as well as DAPI, and subsequently a secondary antibody conjugated to rhodamine was used to visualize the stained E proteins. **(H)** Samples were inspected by fluorescence microscope at a magnification of 200×, and the percentage of E protein-positive cells in six different fields was calculated (the results were expressed as the means and 95% confidence intervals (CIs), and the Mann-Whitney U test was applied for comparisons). **(I)** SNB19 cells were transfected with a specific C19orf66 siRNA or control at a final concentration of 50 nM. After 24 h of transfection, they were challenged with ZIKV at MOI of 1 and harvested 48 hours post infected. The expression levels of C19orf66, ZIKV E and β-actin were analyzed by Western blotting. Prior transfection of SNB19 cells with C19orf66 siRNA increased viral RNA levels in the cells **(J)** and supernatant **(K)**. ZIKV RNA levels were measured by quantitative real time RT-PCR (the data shown in Fig 2J and 2K were expressed as the means ± SDs from three repeat experiments, and were analyzed by one-way ANOVA followed by Dunnett’s multiple comparison test). (**L**) SNB19 cells were transfected with a specific C19orf66 siRNA or negative control at a final concentration of 50 nM. After 24 h of transfection, they were challenged with ZIKV at an MOI of 1, and harvested at the indicated time points post-infection. The number of cellular Zika virus RNA copies was determined by using quantitative real time RT-PCR The data collected at each time point and shown in Fig 2D and 2L were expressed as the means ± SDs from three repeat experiments, and comparisons between two groups at each time point were made by a two-tailed Student’s t test.

To evaluate the role of C19orf66 proteins in ZIKV infection, we generated permanent cell lines stably overexpressing human C19orf66 or control vector, respectively, in hNPC cell line (Fig 2A). The cells were challenged with ZIKV at an MOI of 1, and cellular and supernatant viral RNA were collected and quantified by real time-RT-PCR. Our results showed that C19orf66 overexpression caused a significant reduction of ZIKV RNA quantity, and the fold changes was 0.89±0.04 or 0.18±0.07 respectively (Fig 2B and 2C). Also as shown in the Fig 2D, the viral growth kinetic analysis showed that C19orf66 overexpression caused a significant reduction of ZIKV RNA quantity in a time-dependent manner. Moreover, the role of C19orf66 proteins in ZIKV infection was evaluated by using a plague-forming assay. As shown in Fig 2E and 2F, the plague-forming assay showed a potent inhibitory effect of C19orf66 on the production of ZIKV particles, and PFU ranged from 60.67±3.45×10^3^ /ml to 4.48±1.12×10^3^ /ml. Furthermore, at the protein level, immunoblotting analysis showed that the expression of ZIKV envelop protein (E) was markedly suppressed by C19orf66 overexpression (Fig 2A), and immunofluorescence assays showed the same outcome (Fig 2G and 2H). These results suggest a potent inhibitory effect of C19orf66 on ZIKV RNA and protein synthesis. To further investigate whether endogenous C19orf66 is involved in modulating viral replication, siRNA-mediated knockdown experiments were performed in SNB19 cells. As shown in Fig 2I, 2J and 2K, the depletion of C19orf66 resulted in an increase in ZIKV replication, as indicated by 2.52±0.41 (C19orf66 siRNA-1) and 3.19±0.36 (C19orf66 siRNA-2) fold increases in cellular viral RNA, or 1.87±0.26 (C19orf66 siRNA-1) and 2.55±0.36 (C19orf66 siRNA-2) fold increases in supernatant viral RNA, respectively. Moreover, the depletion of C19orf66 resulted in an increase in the permissiveness of the transfected cells to ZIKV infection in a time-dependent manner (Fig 2L). These results demonstrated that C19orf66 could function to suppress ZIKV propagation.

### C19orf66 colocalizes and interacts with ZIKV NS3

**Fig 3:**
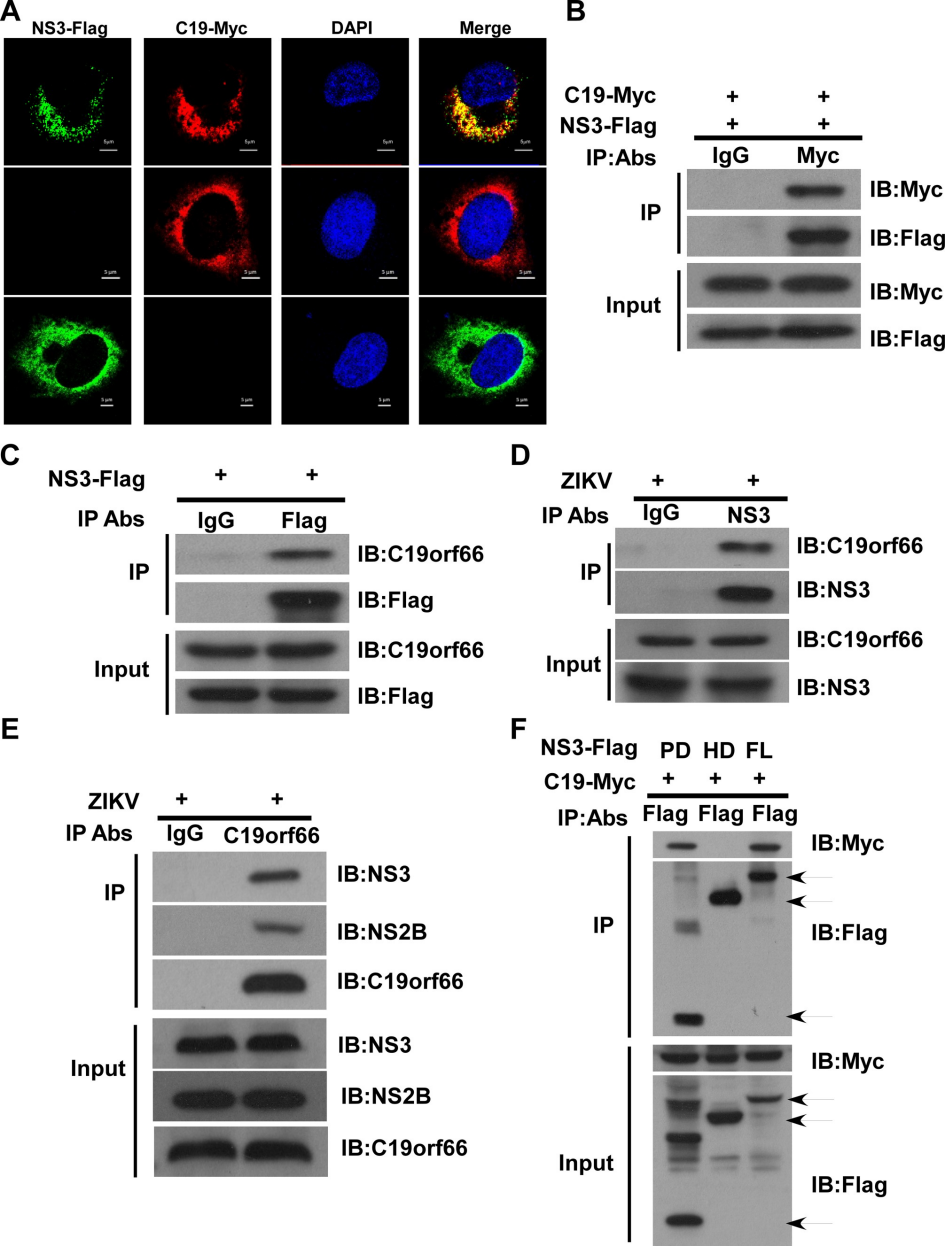
C19orf66 colocalizes and interacts with ZIKV NS3. **(A)** hNPC cells were transiently transfected with a plasmid encoding Flag-tagged ZIKV NS3 or Myc-tagged C19orf66, and 48 hours later, hNPC cells were co-stained with anti-Flag and anti-Myc antibodies as well as DAPI. The co-localization between C19orf66 and ZIKV NS3 is shown in yellow. The data are representative of three independent experiments. **(B)** 293FT cells co-transfected with plasmids encoding Flag-tagged NS3 and Myc-tagged C19orf66 were used for a co-IP assay. Cell lysates were precipitated with an anti-Myc antibody or control IgG, and the immunocomplexes were analyzed with a Flag antibody by Western blotting. **(C)** SNB19 cells were transfected with a plasmid encoding Flag-tagged NS3, followed by immunoprecipitation using anti-Flag antibody or control IgG. The immunocomplexes were analyzed with anti-C19orf66 antibody by Western blotting. **(D)** hNPC cells were infected with ZIKV at MOI of 1, and collected at 48 hours post infection, followed by immunoprecipitation using an anti-NS3 antibody or control IgG. The immunocomplexes were analyzed with anti- C19orf66 antibody by Western blotting. **(E)** hNPC cells were infected with ZIKV at an MOI of 1, and collected at 48 hours post infection, followed by immunoprecipitation using an anti-C19orf66 antibody or control IgG. The immunocomplexes were analyzed with an anti-NS2B or anti-NS3 antibody by Western blotting. **(F)** 293FT cells were co-transfected with plasmids encoding Myc-tagged C19orf66 and individual Flag-tagged truncated NS3 or full-length NS3. At 48 h post-transfection, cells were lysed, co-IP was performed by using an anti-Flag antibody, and the immunocomplexes were analyzed with an anti-Myc antibody by Western blotting.

The previous finding that C19orf66 interacts with DENV NS3 [12], prompted us to ask whether C19orf66 is also able to interfere with ZIKV NS3. To this end, we examined whether NS3 of Zika virus co-localizes with C19orf66 by performing confocal microscopy in cells co-expressing C19orf66-Myc and ZIKV NS3-Flag, after staining with a Flag antibody for NS3 and a Myc antibody for C19orf66 in hNPC cells. As shown in Fig 3A, C19orf66 was found to be co-localized with NS3, supporting the notion that C19orf66 interacts with ZIKV NS3 in host cells.

To confirm a physical interaction between NS3 and C19orf66, we performed co-immunoprecipitation (co-IP) assays after co-transfection of NS3- Flag and C19orf66- Myc expression plasmids in 293FT cells. The co-IP assays showed that C19orf66- Myc was coprecipitated with NS3-Flag (Fig 3B). Our results also showed that C19orf66-Myc was precipitated by the NS3-Flag (S1 Fig), despite that RNase A were also present, suggesting that the interaction between C19orf66 and NS3 is independent of RNA molecules. To better elucidate the interaction between C19orf66 and NS3, we also performed IP to precipitate endogenous C19orf66 using Flag-tagged NS3. Our results showed that endogenous C19orf66 was pulled down together with Flag-tagged NS3 (Fig 3C). Furthermore, to further elucidate the physical interaction between C19orf66 and NS3, we also performed an endogenous IP assay upon ZIKV infection. Our results showed that C19orf66 was precipitated by the NS3 antibody (Fig 3D), meanwhile, ZIKV NS3 or NS2B was precipitated by the C19orf66 antibody (Fig 3E), suggesting that C19orf66 and NS2B/3 form a complex in ZIKV-infected cells. Thus, C19orf66 can bind both ectopic and endogenously expressed ZIKV NS3 protein. Since ZIKV NS3 is a 617-aa protein consisting of two domains, a protease and helicase domains, to identify the NS3 domain(s) required for binding with C19orf66, we generated two deletion mutants, namely NS3-Flag (protease domain, PD)that expressing aa 1 to167, and NS3-Flag (helicase domain, HD)that expressing aa 168 to 617. Co-IP assays using an anti-Flag antibody followed by immunoblotting using an anti-Myc antibody showed that the NS3 protease domain was crucial for its interaction with C19orf66 (Fig 3F).

### C19orf66 expression results in degradation of ZIKV NS3

**Fig 4:**
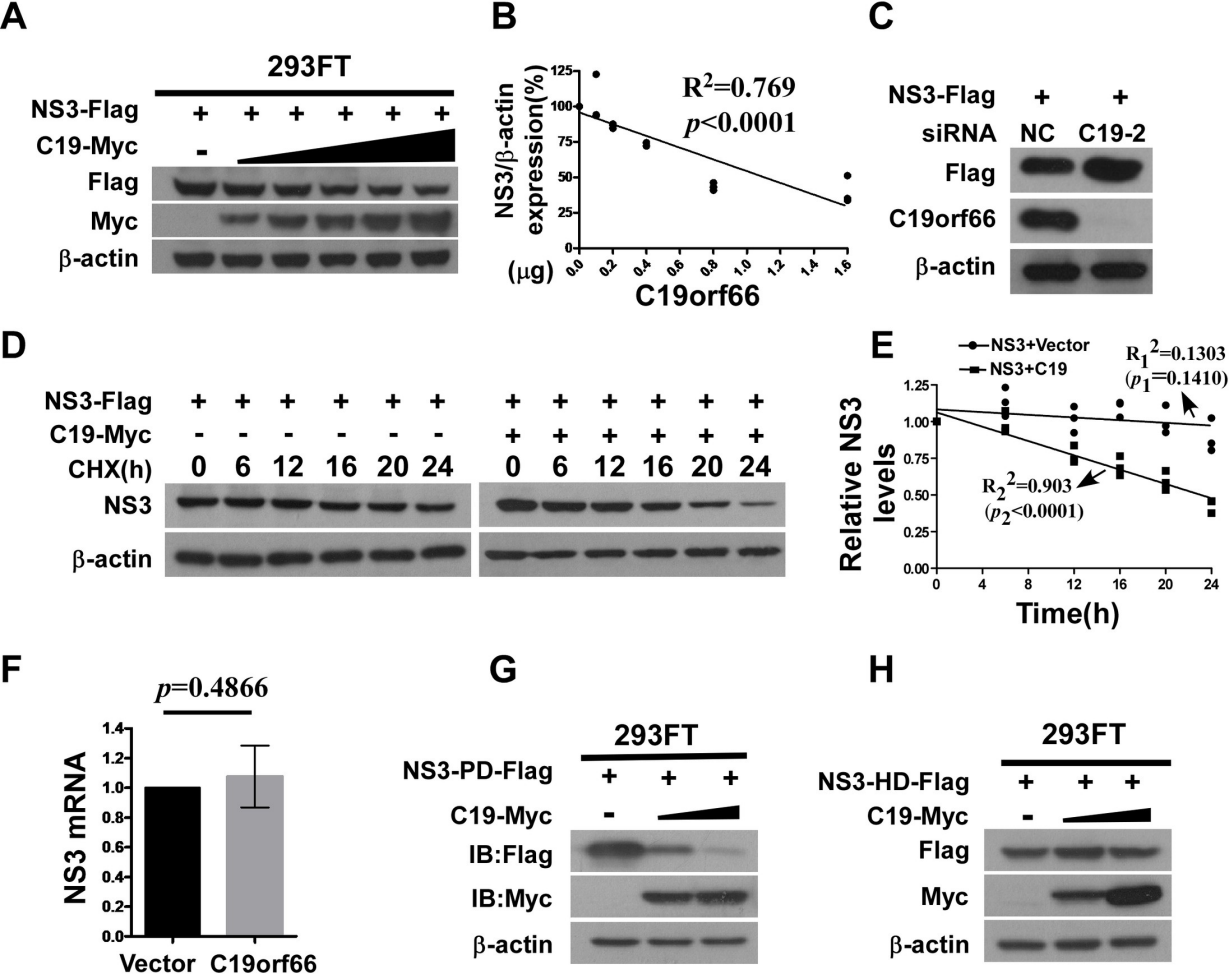
C19orf66 expression results in degradation of ZIKV NS3. **(A)** Western blotting was used to analyze lysates from 293FT cells co-transfected with plasmids encoding ZIKV NS3-Flag and increasing amounts of Myc-C19orf66 (0, 0.1, 0.2, 0.4, 0.8 or 1.6 μg). **(B)** Densitometry analysis of NS3 protein was performed, the percentage of NS3 was calculated, and linear regression analysis was performed to show the relationship between the protein level of C19orf66 and ZIKV NS3 (R^2^ = 0.769, *p*<0.0001). **(C)** SNB19 cells were transfected with a specific C19orf66 siRNA or negative control at a final concentration of 50 nM. After 24 h of transfection, SNB19 cells were transfected a plasmid encoding Flag-tagged NS3, and were harvested at 24 hours. The expression levels of C19orf66, NS3-Flag or β-actin were analyzed by Western Blotting respectively. (**D, E**) 293FT cells were co-transfected with NS3-Flag plasmid and Myc-C19orf66 plasmid or vector plasmid, and 24 h later, the cells were treated with cycloheximide (CHX) (100 μg/ml) for 6, 12, 16, 20, or 24 h. The stability of NS3-Flag was analyzed by Western blotting. The protein levels of NS3 or C19orf66 were normalized to the level of β-actin using band intensity values. The half-life (t1/2) of NS3 co-transfected with Myc-C19orf66 was determined by linear regression analysis and calculated by the following formula, y = -0.024x+1.063 (R_1_^2^ = 0.903, *p*_1_<0.0001). The group that NS3 transfected without Myc-C19orf66 was analysis with linear regression analysis, and got the following formula, y = -0.005x+1.083 (R_2_^2^ = 0.1303, *p*_2_ = 0.1410). **(F)** Quantitative real time RT-PCR was used to analyze NS3 mRNA expression in 293FT cells 36 hours after co-transfection with NS3-Flag (1.6 μg) and with Myc-C19orf66 (1.6 μg) or control plasmid (the results were expressed as the means and 95% CIs from three repeat experiments, and comparisons were made by the Mann-Whitney U test). (**G**, **H**) Western blot analysis of lysates from 293FT cells co-transfected with C19orf66 and the NS3-deletion constructs, (**G**) protease domain (PD), or (**H**) helicase domain (HD).

Antiviral proteins that mediate the degradation of viral proteins are one of mechanisms of host defense. As we found that C19orf66 interacts with ZIKV NS3, we next asked whether C19orf66 could promote the degradation of ZIKV NS3. To test this, increasing amounts of C19orf66 expression plasmid along with a plasmid expressing NS3 were transfected into cells. Proteins were expressed for 48 h and analyzed by Western blotting followed by quantification of the bands. The results showed that there was a dose-dependent reduction in NS3 protein levels (Fig 4A and 4B). To further investigate whether endogenous C19orf66 is involved in reducing NS3, siRNA-mediated knockdown experiments were performed in SNB19 cells that constitutively express high basal levels of endogenous C19orf66 expression. As shown in Fig 4C, the depletion of C19orf66 resulted in an increase in the expression level of NS3. The reduction in ZIKV NS3-Flag proteins by C19orf66 might be caused by the inhibition of plasmid encoded ZIKV NS3-Flag transcription. To assess this possibility, a plasmid encoded C19orf66-Myc or control plasmids were co-transfected with NS3-Flag into 293FT cells, and the abundance of NS3-Flag mRNA was measured by quantitative real time PCR. We found that C19orf66-Myc overexpression had no impact on NS3-Flag mRNA abundance (Fig 4F), suggesting that the effect of C19orf66-Myc in mediating NS3 degradation was not caused by suppressing the transcription of plasmids encoding NS3-Flag. Furthermore, the half-life (t1/2) of NS3 was determined by linear regression analysis. As shown in Fig 4D and 4E, the half-life of NS3 was 23.45 h when NS3 was co-expressed with C19orf66, and the half-life of NS3 alone was longer than 24 h when NS3 was expressed alone, suggesting that NS3 had a shorter half-life while interacted with C19orf66. Moreover, the protease domain (PD) of NS3 was markedly reduced in the C19orf66-Myc-expressing cells (Fig 4G), but not the helicase domain (HD) of NS3 (Fig 4H), which is consistent with the interaction between C19orf66 and the NS3 protease domain. These data indicated that the stability of ZIKV NS3 was affected by C19orf66 expression.

### C19orf66 mediates ZIKV NS3 protein degradation through the lysosomal pathway

**Fig 5:**
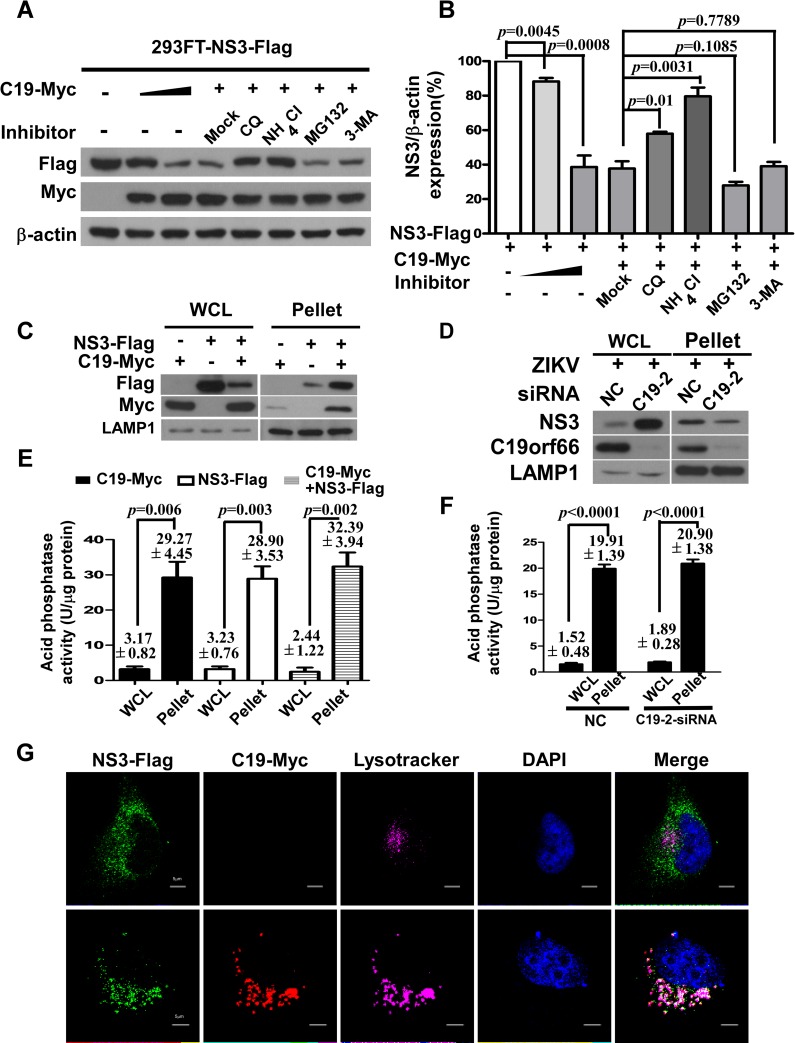
C19orf66 influences ZIKV NS3 protein degradation through the lysosome pathway. **(A, B)** 293FT cells were co-transfected with plasmids encoding Flag-tagged NS3 and Myc-tagged C19orf66, 36 hours later, the cells were treated with CQ (5 μM), NH_4_Cl (2 mM), MG132 (5 μM) or 3-MA (5 mM). Densitometry analysis of NS3 protein was performed, and the percentage of NS3 was calculated (the data were expressed as the means ± SDs from three repeat experiments, and comparisons between two groups were made by a two-tailed Student’s t test). (**C**) 293FT cells were transfected with an NS3-Flag, or C19orf66-Myc plasmids alone, or co-transfected with NS3- Flag and C19orf66-Myc plasmids for 24 h. (**D**) SNB19 cells were transfected with a specific C19orf66 siRNA or negative control (NC) for 24 h, infected with ZIKV at an MOI of 1 and harvested 48 hours post-infection. Lysosomes were isolated from cells while transfected with or without NS3 by the Lysosome Enrichment Kit. The lysosomal lysate was also analyzed by Western blotting analysis for C19orf66, NS3, and LAMP1 (a lysosome marker). (**E, F**) To confirm that the extract was the lysosomal fraction, we assessed the activity of acid phosphatase, which is a kind of lysosome marker enzyme (the results were expressed as the means ± SDs from three repeat experiments, and comparisons between two groups were made by a two-tailed Student’s t test). **(G)** hNPC cells were transfected with a plasmid encoding Flag-tagged NS3, with or without Myc C19orf66. The lysosomes, NS3, C19orf66 and nuclei were co-stained using LysoTracker (magenta), a Flag antibody (green), a Myc antibody (red) and DAPI (blue). The cells were analyzed using fluorescence microscopy.

We then proposed whether C19orf66 triggers NS3 degradation via the proteasomal pathway. When 293FT cells were co-transfected with Myc-tagged C19orf66 and Flag-tagged NS3, treated with proteasomal inhibitor MG132 at one of two doses (10 μM or 20 μM), and harvested at various indicated times, immunoblot analysis showed that the abundance of NS3 was not restored (S2A and S2B Fig). Next, we found that the co-expression of C19orf66 and NS3 did not up-regulate the polyubiquitination level of the NS3 protein in 293FT cells, even after treatment with MG132 (S2C Fig). These results suggested that the C19orf66-induced degradation of NS3 was independent of the proteasomal pathway. To determine the cellular machinery responsible for NS3 degradation by C19orf66, the lysosomal inhibitor ammonium chloride (NH_4_Cl), chloroquine diphosphate (CQ), and the autophagy inhibitor 3-methyladenine (3-MA) were used to examine the inhibitory effects. Interestingly, the results showed that the treatments with CQ and NH_4_Cl significantly reversed NS3 degradation mediated by C19orf66, but not MG132 or 3-MA (Fig 5A and 5B), and that the percentages of NS3 expression compared to the control (without inhibitor treatment and without C19-Myc over-expression), for each group was as following: 88.29±3.52% (C19-Myc over-expression group, 0.1 μg of plasmid), 38.59±11.61% (C19-Myc over-expression group, 1.6 μg of plasmid), 37.68±7.36% (Mock), 57.96±2.00% (CQ), 79.66±8.73% (NH_4_Cl), 27.92±3.64% (MG132), or 39.14±4.12% (3-MA) respectively. Meanwhile, we detected the inhibition of the proteasome pathway by monitoring the total protein level of p65 in 293FT cells under treatment with MG132. We also further examined the induction of autophagy by Western blotting for the level of the autophagy marker protein light chain 3 (LC3) in 293FT cells under treatment with 3-MA, which converts the soluble form of LC3-I to the lipidated form of LC3-II and serves as an indicator of autophagy. As shown in S3 Fig, immunoblot analysis showed that the expression of p65 increased in cells exposed to MG132 for 6 h, and that LC3-II was increased in cells exposed to 3-MA for 6 h, respectively, suggesting the drugs had the expected effects.

The localization of NS3 in purified lysosomes in the presence or absence of C19orf66 in cells that infected with ZIKV, was further evaluated by a lysosome enrichment assay. Lysosomes were isolated from ZIKV-infected SNB19 cells by the Lysosome Enrichment Kit, followed analyzed by Western blotting for C19orf66, NS3, and LAMP1 (a lysosomal marker). As shown in Fig 5C and S4A and S4B Fig, we found that the NS3 level in the lysosomal pellet of cells co-transfected with C19orf66 and NS3, was higher than that of in the lysosomal pellet of cells transfected with NS3 alone. Conversely, the results showed that the NS3 level in the lysosomal pellet of cells while depletion of C19orf66, was lower than that in the lysosomal pellet of wild-type cells when infected with ZIKV (Fig 5D), suggesting that the lysosomal degradation of NS3 might be dependent on C19orf66. To confirm that the extract was the lysosomal fractions, we assessed the activity of acid phosphatase, which is a kind of lysosome-specific enzyme. The assessment of the total cell lysate and lysosome-enriched fractions demonstrated that the lysosome-enriched fraction possessed approximately20-fold greater acid phosphatase activity than the total lysate (Fig 5E and 5F). Moreover, our data suggested that the inhibition of the lysosome did not increase NS3 expression in the absence of C19orf66 (S5 Fig). While the ZIKV protein NS2B/3 can interact with and cleave STING, resulting in attenuated IFN-I production. To further elucidate that the NS3-C19orf66 interaction can prevent NS3 protease activity, we also performed Western blotting to examine the cleavage of STING upon co-transfection with C19orf66 and NS3 in hNPC cells. Our data showed that the cleavage of STING by NS2B/3 was blocked by C19orf66 (S6 Fig). To further confirm that NS3 is degraded in a lysosome-dependent manner, we performed immunofluorescence assays to visualize the localization of NS3 in lysosomes. As shown in the S4C Fig, we found that C19orf66 was rarely localized in lysosomes in the absence of NS3. And the results showed that the co-localization of NS3 in lysosomes was detected using LysoTracker and an anti-Flag antibody, and that C19orf66 increased the co-localization (Fig 5G). In summary, these data demonstrate that C19orf66 promotes the degradation of ZIKV NS3 proteins through the lysosome pathway.

## Discussion

The recent epidemic of ZIKV and its link to severe complications, including fetal abnormalities, microcephaly, neurological autoimmune disorder and Guillain-Barré syndrome, have created a serious threat to global health [1]. Type I interferons exert their immune regulatory and protective roles through the coordinate expression of hundreds of ISGs, which ultimately rendering the cell to establish an antiviral state against viral infection, including ZIKV [24]. In the present study, we revealed the functional properties of a novel IFN-I inducible protein termed C19orf66 in response to ZIKV infection. Our work identified C19orf66 as a potent restriction host factor for ZIKV infection via interacting with the NS3 protein and modulating its stability. We found that C19orf66 could bind both ectopic and endogenously expressed ZIKV NS3 protein, which might suppress the process of NS3-mediated the viral polyprotein cleavage. Our results further demonstrated that C19orf66 promotes NS3 degradation through the lysosome pathway as the degradation procedure of NS3 that induced by C19orf66 could be reversed by treatment with the lysosome inhibitors-chloroquine or NH_4_Cl, but not MG132 (an inhibitor of the proteasome pathway), or 3-MA (an autophagosome-formation inhibitor).

C19orf66 was first suggested to inhibit several important viruses in a study by using an overexpression screening approach [25]. C19orf66 has been shown to combat the replication of DENV, HCV, Chikungunya virus, herpes simplex virus type 1, and human adenovirus [12, 13]. Consistent with current reports, we also found that C19orf66 could inhibit the multiplication of influenza virus (S7 Fig). The findings in the present study have shown that the loss of C19orf66 expression renders cells more susceptible to ZIKV replication, thereby adding ZIKV to that list, and proposing that C19orf66 is critical to the antiviral response against a wide variety of viruses through differences underlying mechanism. Accordingly, experiments using C19orf66-deficient mice are needed to address the *in vivo* significance of C19orf66 in antiviral immune responses, which is an issue worth of further investigation. Our model system could potentially be used to uncover the specific targets and mechanisms underlying the antiviral functions of C19orf66 against other related flaviviruses, such as DENV, or WNV. Of note, it was reported that C19orf66 interfered with the translation of DENV via binding with viral RNA, positive modulators PABPC1 and LARP1, leading to the inhibition of viral replication in cells [12]. Meanwhile, Balinsky and colleagues found that C19orf66 co-localized with cytoplasmic processing bodies (P bodies) in cells, where it interacts with the MOV10 RISC complex RNA helicase and restricted DENV through influencing the processing of DENV viral RNA [13]. It was noted that the ZIKV NS3 protease domain is important for viral polyprotein cleavage, and reducing type I IFN production by cleaving the adaptor protein STING [7]. Interestingly, we here found that C19orf66 targeted the NS3 protease domain. We also observed that the cleavage of STING induced by NS2B3 is blocked by C19orf66. Hypothetically, it would be of great interest to further identify whether C19orf66 prevents ZIKV evading the innate immune response through STING-dependent type I interferon production. These processes may represent other means through which C19orf66 inhibits viral replication, and it would be of great interest to elaborate the function of C19orf66 on the replication or translation of ZIKV through interactions with PABPC1, LARP1, or ZIKV replication complex. Together, our work highlights diverse antiviral mechanisms of C19orf66 and suggests that C19orf66 agonists may have clinical utility when repurposed as ZIKV treatments.

Here, we show that C19orf66 mediates NS3 protein degradation in a lysosome-dependent manner to suppress ZIKV replication. Autophagy is a catabolic process in maintaining tissue, organism, and cell homeostasis through the degradation of cellular contents, including proteins and organelles using autophagic vacuoles [26, 27]. There are at least three types of autophagy in mammals, including macroautophagy, microautophagy, and chaperone-mediated autophagy [26, 27]. Although these three protein degradation systems are mediated by different mechanisms and different molecules, they are categorized as autophagy because lysosomes are involved in their proteolytic process [27]. Lysosomes are known primarily to degrade macromolecules or infectious pathogens from part of three major cellular degradative pathways: autophagy and endocytosis, and phagocytosis, which are essential for innate immunity recognition, antigen presentation, and pathogen elimination. Lysosomes degrade extracellular material that has been internalized by endocytosis, and intracellular components that have been sequestered by autophagy [27]. It has been reported that ZIKV potently induces autophagy for efficient viral replication and virion assembly [28]. In this work, we found that treatments with chloroquine and ammonium chloride (NH_4_Cl) (lysosomal acidification inhibitors), but not 3-MA (an autophagosome-formation inhibitor) significantly reversed NS3 degradation mediating by C19orf66. It was reported that the chaperone-mediated autophagy (CMA) motifs have been used to target cytosolic proteins including huntingtin27, DAPK1, DLG4 and α-synuclein, which are not affected by 3-MA [29, 30]. Also, Jie Xu *et al* reported that Huntingtin-interacting protein 1-related protein (HIP1R) was involved in mediating lysosomal degradation of PD-L1, but the inhibitors for autophagy (3-MA) displayed no effect on HIP1R [31]. Thus, it might be important to further investigate which stage of lysosome-mediated degradative pathways, i.e., endocytic, autophagic, or phagocytic pathways, is influenced by C19orf66, and which lysosome-related molecules interact with C19orf66 to mediate NS3 degradation.

In summary, the current findings reveal host innate immunity against ZIKV and the possible underlying molecular mechanisms. Our study found that C19orf66 restricted ZIKV replication via mediating NS3 degradation in the lysosome-dependent pathway. This work not only identified a novel ISG, C19orf66, that protected against ZIKV, but also unveiled a novel antiviral mechanism of C19orf66, thereby offering a new therapeutic agent for ZIKV infection.

## Supporting information

S1 Table Sequences of primers used in this work. (DOCX)

S1 Fig The interaction between C19orf66 and NS3 is independent of RNA. 293FT cells co-transfected with plasmids encoding Flag-tagged NS3 and Myc-tagged C19orf66 were used in a co-IP assay. Cell lysates treated with or without RNases (10 μg/μl), were precipitated with an anti-Flag antibody or control IgG, and the immunocomplexes were analyzed with an anti-Myc antibody by Western blotting.
(TIF)

S2 Fig C19orf66-induced degradation of NS3 is independent of the proteasomal pathway. (**A**) 293FT cells were cotransfected with plasmids encoding Flag-tagged NS3 and Myc-tagged C19orf66,36 hours later, the cells were treated with MG-132 at different concentration (10 μM, or 20 μM) for 6 hours, and the protein expression levels were detected by Western blotting. (**B**) 293FT cells were cotransfected with plasmids encoding Flag-tagged NS3 and Myc-tagged C19orf66, then cells were treated with MG132, and 3, 6, 9, and 12 hours post treatment, the protein expression levels were detected by Western blotting. (**C**) 293FT cells were cotransfected with plasmids encoding Flag-tagged NS3, Myc-tagged C19orf66 and HA-tagged ubiquitin, MG132 treatment was performed 4 h before the total protein was extracted. Co-IP was performed by using an anti-Flag antibody, and the immunocomplexes were analyzed by Western blotting using an anti-HA antibody.
(TIF)

S3 Fig The positive control of inhibitors MG132 and 3-MA. The inhibition of the proteasome pathway was detected by monitoring the total protein level of p65 in 293FT cells under treatment with MG132. Moreover, the induction of autophagy was further examined by Western blotting for the level of the autophagy marker protein light chain 3 (LC3) in 293FT cells under treatment with 3-MA, which converts the soluble form LC3-I to the lipidated form LC3-II and serves as an indicator of autophagy. Western blotting was used to analyze lysates from 293FT cells that treated with 10 μM MG132 (**A**) or 5 mM 3-MA (**B**) for 6 hours.
(TIF)

S4 Fig C19orf66 influences ZIKV NS3 protein degradation through the lysosome pathway. (**A**) 293FT cells were transfected with an NS3-Flag, or C19orf66-Myc plasmid alone, or co-transfected with NS3- Flag and C19orf66-Myc plasmids for 24 h. (**B**) SNB19 cells were transfected with a specific C19orf66 siRNA or negative control (NC) for 24 h, and infected with ZIKV at an MOI of 1, and the supernatant was harvested 48 hours post infection. The supernatant was also analyzed by Western blotting analysis for C19orf66, NS3, and LAMP1 (a lysosomal marker). (**C**) hNPC cells were transfected with a plasmid encoding Myc C19orf66. The lysosomes, NS3, C19orf66 and nucleus were co-stained with LysoTracker (magenta), an anti-Flag antibody (green), an anti-Myc antibody (red) and DAPI (blue). The cells were analyzed using fluorescence microscopy.
(TIF)

S5 Fig Inhibition of lysosomes does not increase NS3 expression in the absence of C19orf66. hNPC cells were transfected with a siRNA negative control at a final concentration of 50 nM. After 24 h transfection, hNPC cells were transfected with a plasmid encoding Flag-tagged NS3, followed by treated with or without CQ (5μM), and harvested after 24 hours. The expression levels of C19orf66, Flag-NS3 and β-actin were analyzed by Western blotting.
(TIF)

S6 Fig The cleavage of STING by NS2B/3 is blocked by C19orf66. hNPC cells were transfected with plasmids encoding HA-tagged STING alone, or co-transfected with His-tagged NS2B/3, or co-transfected with both His-tagged NS2B/3 and Myc-tagged C19orf66, and cells were harvested at 48 hours. The expression levels of HA-STING, His- NS2B/3, Myc-C19orf66 and β-actin were analyzed by Western blotting.
(TIF)

S7 Fig C19orf66 restricts influenza A virus infection in cells. A549 cells were stably transduced with retrovirus vector expressing C19orf66 or vector control, infected with influenza A virus (H1N1) at an MOI of 0.5, and then collected 48 hours post infection. The indicated cellular viral RNA (**A**) and supernatant viral RNA (**B**) levels were determined by using real time RT-PCR. The expression levels were normalized to the level of GAPDH. The results were expressed as the means ± SDs from three repeat experiments, and comparisons were evaluated by a two-tailed Student’s t test.
(TIF)
