# Supplementary material for: C19orf66 interrupts Zika virus replication by inducing lysosomal degradation of viral NS3
Source: PLoS Negl Trop Dis. 2020 Mar 9;14(3):e0008083. doi: 10.1371/journal.pntd.0008083 (PMC7082052; doi:10.1371/journal.pntd.0008083)
Supplement: S1 Table — (DOCX) [file pntd.0008083.s001.docx]

**Supplemental** **Table S1 Sequences of primers used in this work**

|  | Primers | Sequence (5’-3’) |
| --- | --- | --- |
| Myc-C19orf66 | Forward | CGGGGTACCGCCATGTCTCAGGAAGGTGTGGAGCTGG |
|  | Reverse | TGCTCTAGACTACAGATCTTCTTCAGAAATAAGTTTTTGTTCCTCCCTGGGCCCGCCCTC |
| Flag-NS3 | Forward | CGGGGTACCGCCATGGATTACAAGGATGACGACGATAAG AGTGGTGCTCTATGGGATG |
|  | Reverse | TGCTCTAGACTATCTTTTCCCAGCGGCAAAC |
| Flag-NS3(1-170) | Forward | CGGGGTACCGCCATGGATTACAAGGATGACGACGATAAGAGTGGTGCTCTATGGGATG |
|  | Reverse | TGCTCTAGATCACCTCCTCCCTTGGGTGATG |
| Flag-NS3(171-617) | Forward | CGGGGTACCGCCATGGATTACAAGGATGACGACGATAAGGAGGAAGAGACTCCTGTTG |
|  | Reverse | TGCTCTAGATCATCTTTTCCCAGCGGCAAAC |
| hC19orf66 | Forward | AGTAACGATCTGGATGCCCA |
|  | Reverse | ACATGCGTAGGTTGGCTTCT |
| mC19orf66 | Forward | CTCAGGATGGCGTGGA |
|  | Reverse | TCACTCTCGGGGCCCACCGT |
| NS3 | Forward | GTTTGGCTGGCCTATCAGGT |
|  | Reverse | CACCTCGGTTTGAGCACTCT |
| ZIKV | Forward | CCGCTGCCCAACACAAG |
|  | Reverse | CCACTAACGTTCTTTTGCAGACAT |
|  | Probe | AGCCTACCTTGACAAGCAGTCAGACACTCAA |
| GAPDH | Forward | GACTCATGACCACAGTCCATGC |
|  | Reverse | AGAGGCAGGGATGATGTTCTG |
|  | Probe | CATCACTGCCACCCAGAAGACTGTG |
| mGAPDH | Forward | GGCAAATTCAACGGCACAGT |
|  | Reverse | GGGTCTCGCTCCTGGAAGAT |
|  | Probe | AAGGCCGAGAATGGGAAGCTTGTCATC |
| siRNA-C19orf66-1 |  | GCCAAGAACTAAGTAACGA |
| siRNA-C19orf66-2 |  | CCAACCTACGCATGTTTCA |
