# Supplementary figures and images for: C19orf66 interrupts Zika virus replication by inducing lysosomal degradation of viral NS3
Source: PLoS Negl Trop Dis. 2020 Mar 9;14(3):e0008083. doi: 10.1371/journal.pntd.0008083 (PMC7082052; doi:10.1371/journal.pntd.0008083)

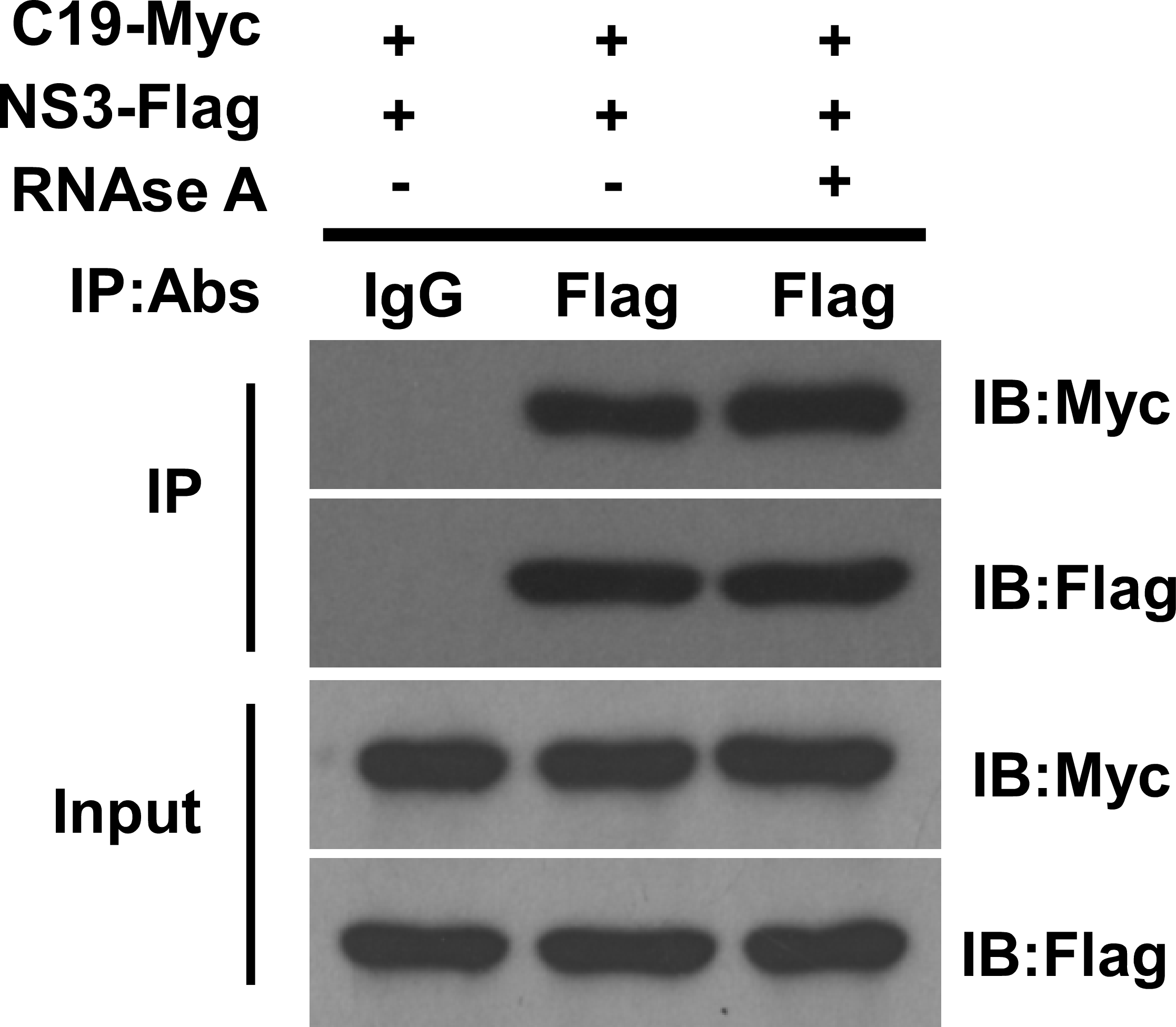

Supplement: S1 Fig — 293FT cells co-transfected with plasmids encoding Flag-tagged NS3 and Myc-tagged C19orf66 were used in a co-IP assay. Cell lysates treated with or without RNases (10 μg/μl), were precipitated with an anti-Flag antibody or control IgG, and the immunocomplexes were analyzed with an anti-Myc antibody by Western blotting. (TIF) [file pntd.0008083.s002.tif]

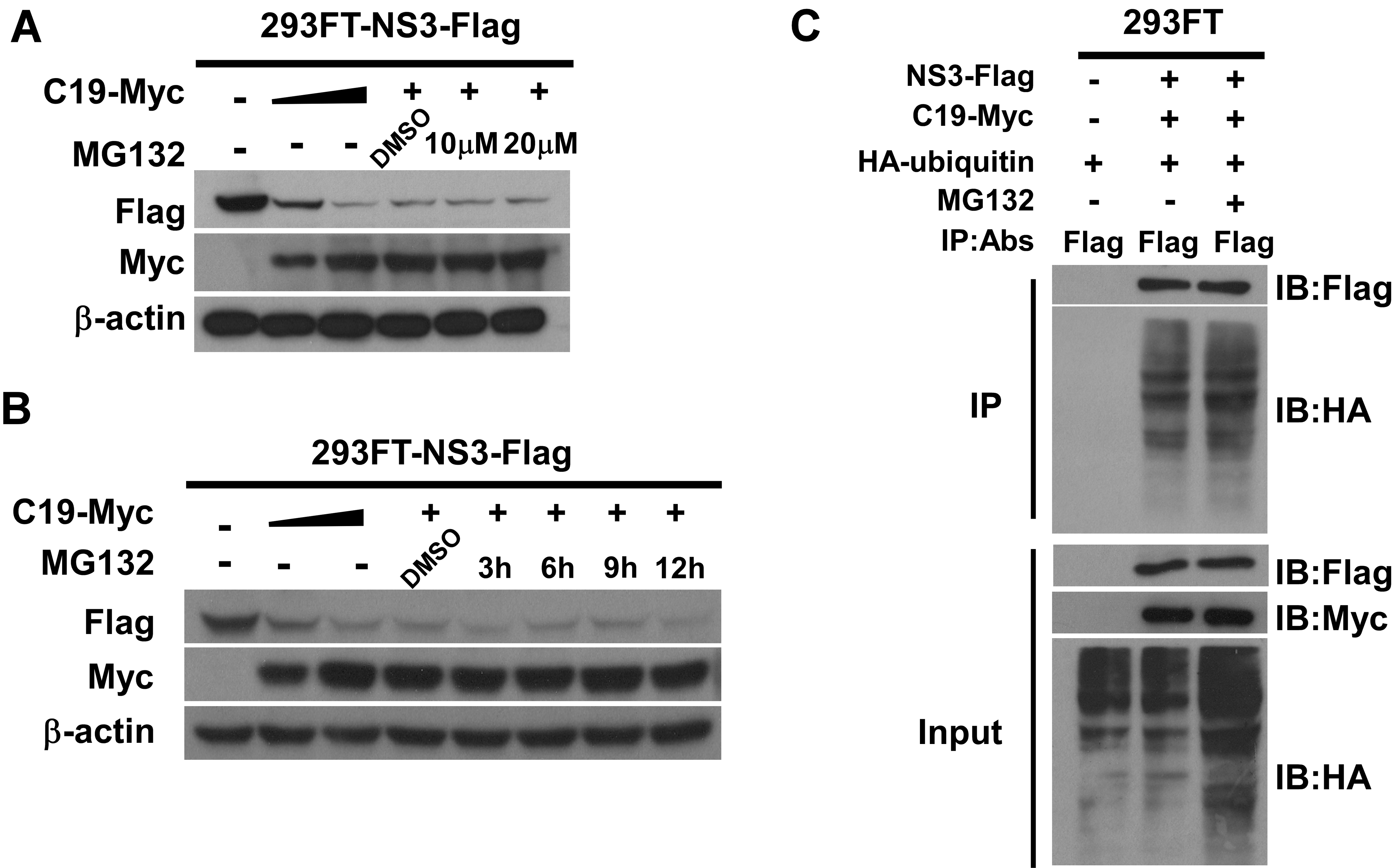

Supplement: S2 Fig — (A) 293FT cells were cotransfected with plasmids encoding Flag-tagged NS3 and Myc-tagged C19orf66,36 hours later, the cells were treated with MG-132 at different concentration (10 μM, or 20 μM) for 6 hours, and the protein expression levels were detected by Western blotting. (B) 293FT cells were cotransfected with plasmids encoding Flag-tagged NS3 and Myc-tagged C19orf66, then cells were treated with MG132, and 3, 6, 9, and 12 hours post treatment, the protein expression levels were detected by Western blotting. (C) 293FT cells were cotransfected with plasmids encoding Flag-tagged NS3, Myc-tagged C19orf66 and HA-tagged ubiquitin, MG132 treatment was performed 4 h before the total protein was extracted. Co-IP was performed by using an anti-Flag antibody, and the immunocomplexes were analyzed by Western blotting using an anti-HA antibody. (TIF) [file pntd.0008083.s003.tif]

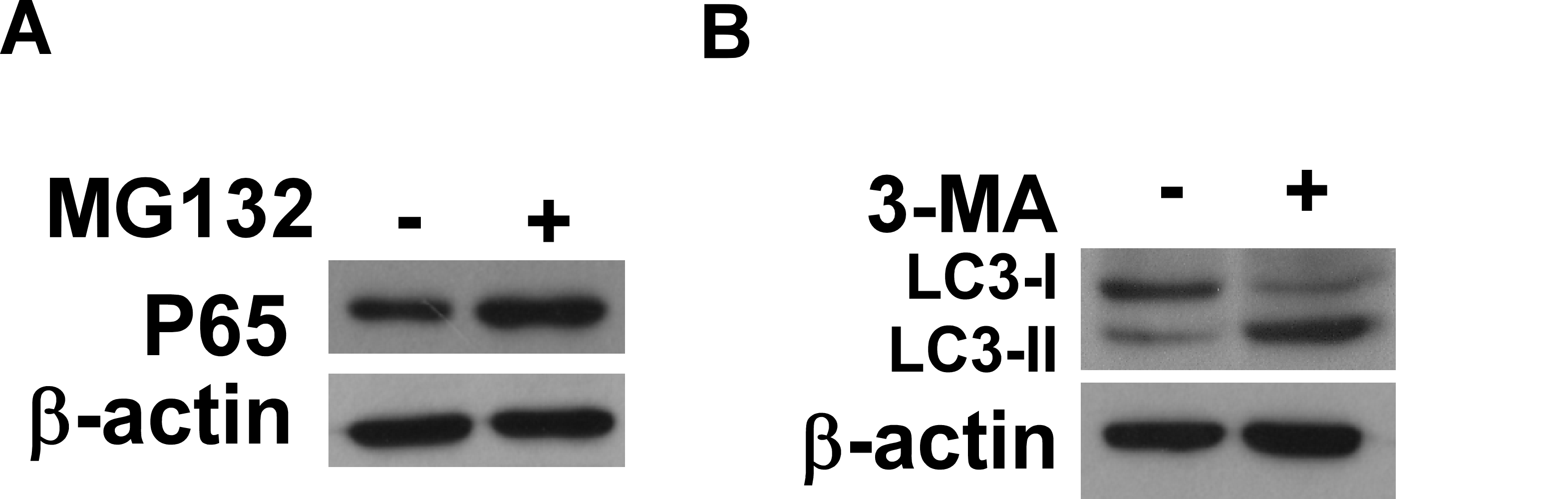

Supplement: S3 Fig — The inhibition of the proteasome pathway was detected by monitoring the total protein level of p65 in 293FT cells under treatment with MG132. Moreover, the induction of autophagy was further examined by Western blotting for the level of the autophagy marker protein light chain 3 (LC3) in 293FT cells under treatment with 3-MA, which converts the soluble form LC3-I to the lipidated form LC3-II and serves as an indicator of autophagy. Western blotting was used to analyze lysates from 293FT cells that treated with 10 μM MG132 (A) or 5 mM 3-MA (B) for 6 hours. (TIF) [file pntd.0008083.s004.tif]

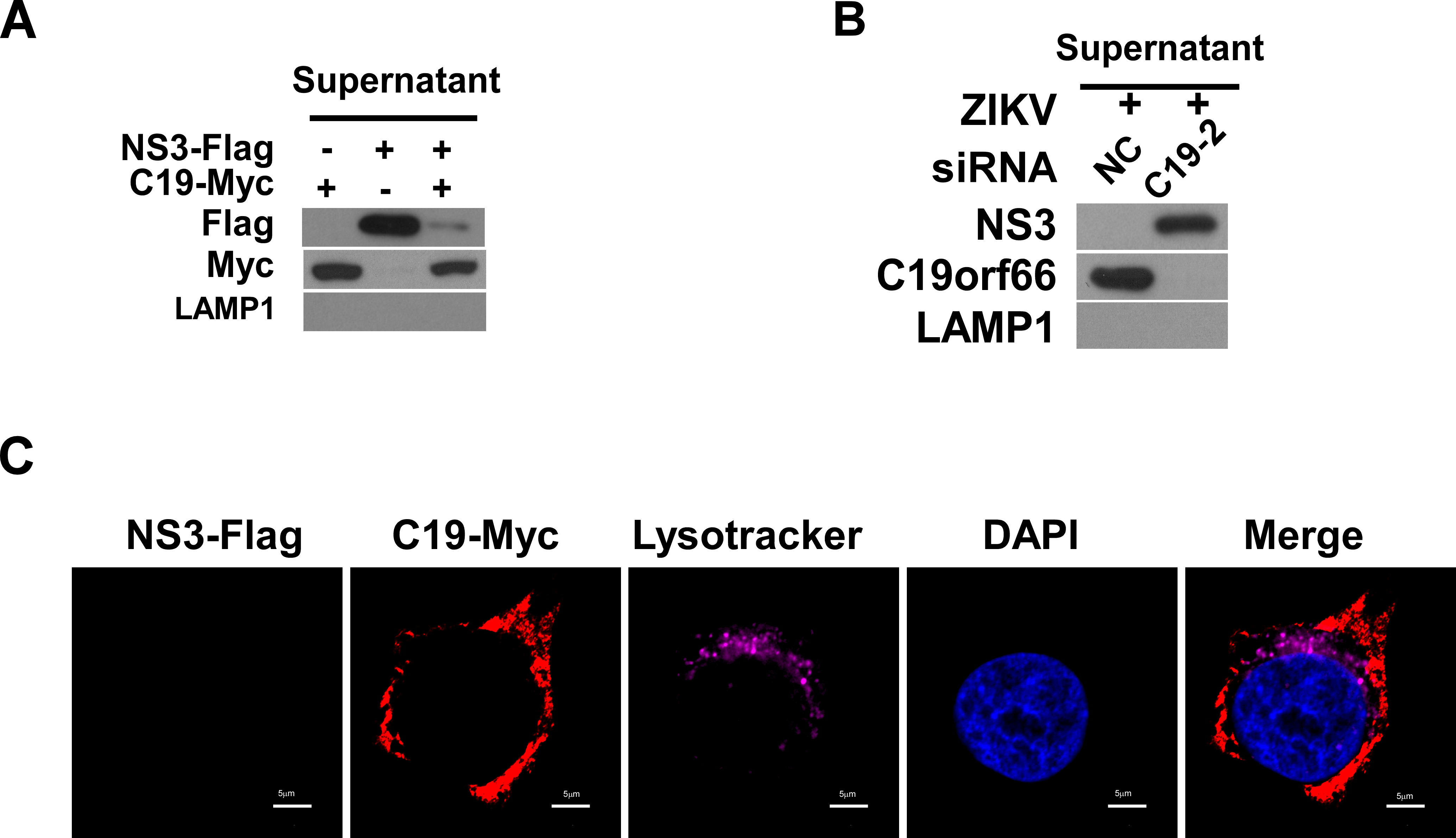

Supplement: S4 Fig — (A) 293FT cells were transfected with an NS3-Flag, or C19orf66-Myc plasmid alone, or co-transfected with NS3- Flag and C19orf66-Myc plasmids for 24 h. (B) SNB19 cells were transfected with a specific C19orf66 siRNA or negative control (NC) for 24 h, and infected with ZIKV at an MOI of 1, and the supernatant was harvested 48 hours post infection. The supernatant was also analyzed by Western blotting analysis for C19orf66, NS3, and LAMP1 (a lysosomal marker). (C) hNPC cells were transfected with a plasmid encoding Myc C19orf66. The lysosomes, NS3, C19orf66 and nucleus were co-stained with LysoTracker (magenta), an anti-Flag antibody (green), an anti-Myc antibody (red) and DAPI (blue). The cells were analyzed using fluorescence microscopy. (TIF) [file pntd.0008083.s005.tif]

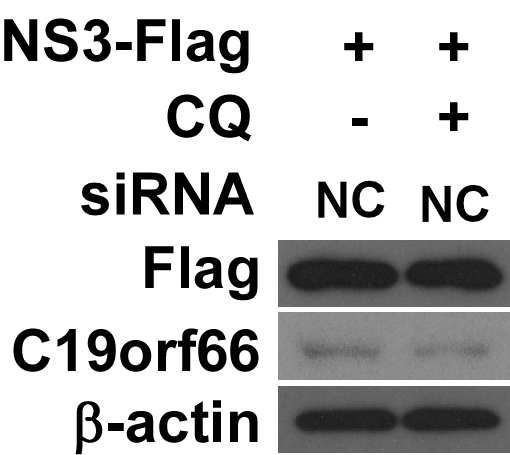

Supplement: S5 Fig — hNPC cells were transfected with a siRNA negative control at a final concentration of 50 nM. After 24 h transfection, hNPC cells were transfected with a plasmid encoding Flag-tagged NS3, followed by treated with or without CQ (5μM), and harvested after 24 hours. The expression levels of C19orf66, Flag-NS3 and β-actin were analyzed by Western blotting. (TIF) [file pntd.0008083.s006.tif]

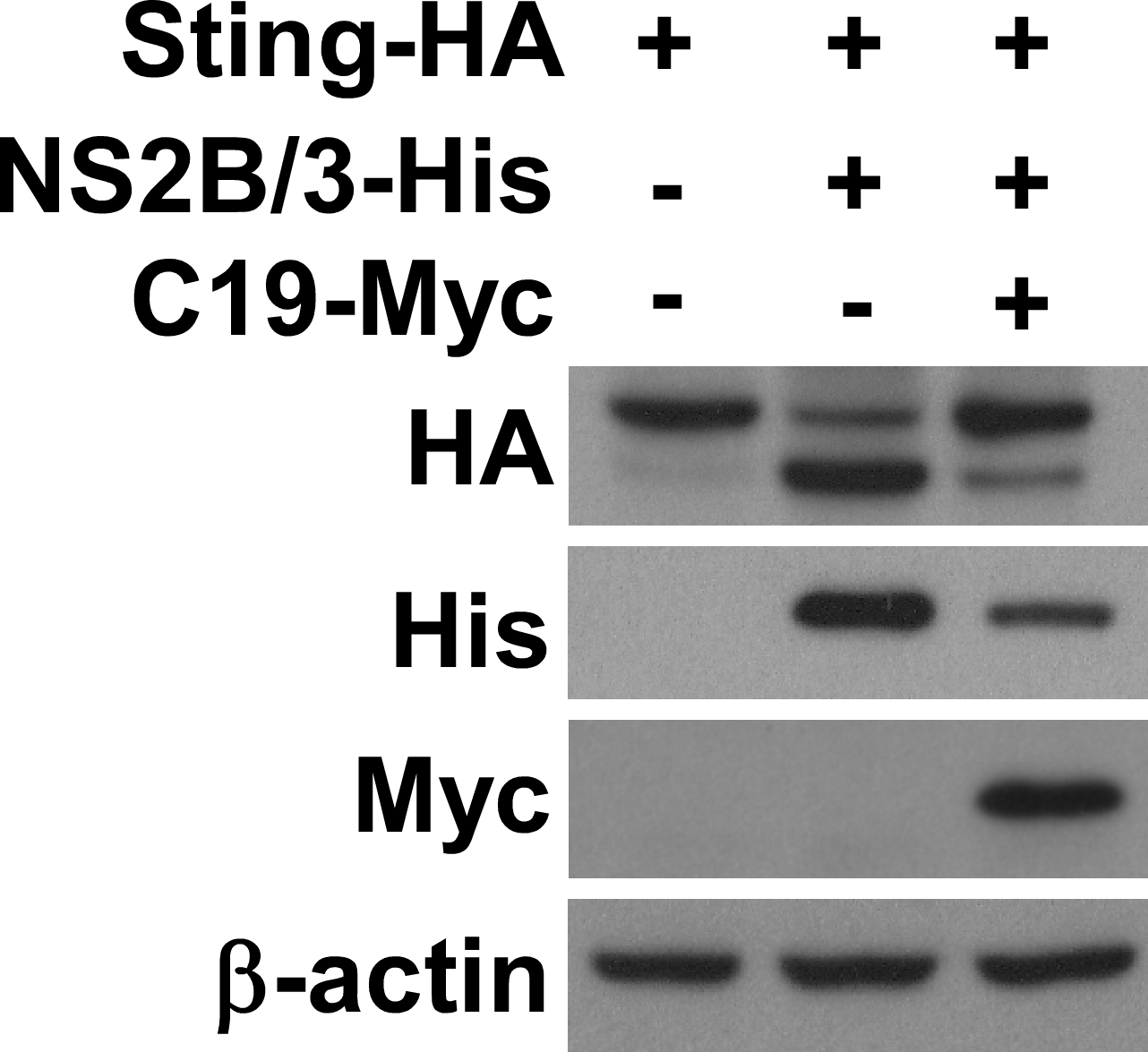

Supplement: S6 Fig — hNPC cells were transfected with plasmids encoding HA-tagged STING alone, or co-transfected with His-tagged NS2B/3, or co-transfected with both His-tagged NS2B/3 and Myc-tagged C19orf66, and cells were harvested at 48 hours. The expression levels of HA-STING, His- NS2B/3, Myc-C19orf66 and β-actin were analyzed by Western blotting. (TIF) [file pntd.0008083.s007.tif]

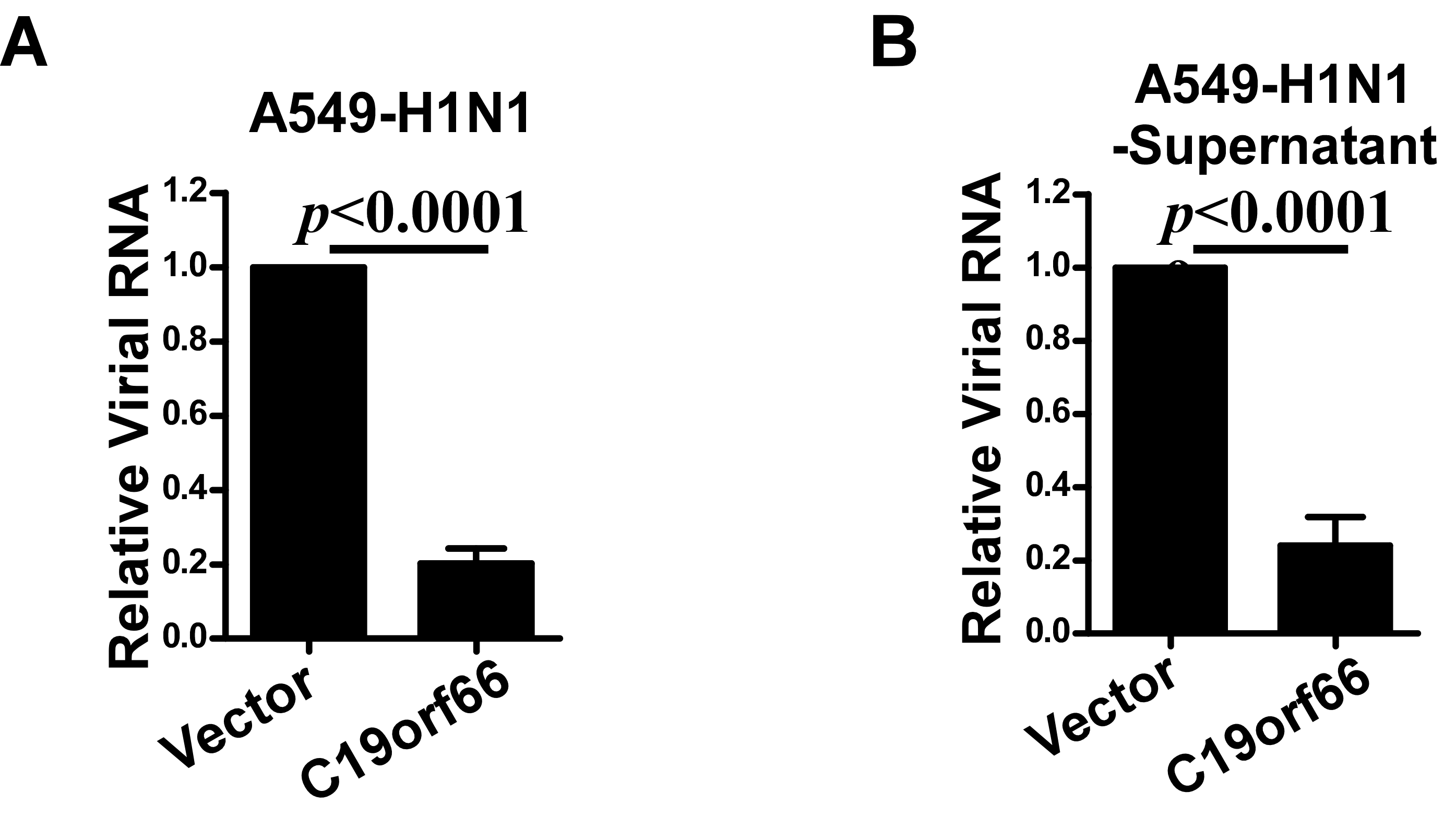

Supplement: S7 Fig — A549 cells were stably transduced with retrovirus vector expressing C19orf66 or vector control, infected with influenza A virus (H1N1) at an MOI of 0.5, and then collected 48 hours post infection. The indicated cellular viral RNA (A) and supernatant viral RNA (B) levels were determined by using real time RT-PCR. The expression levels were normalized to the level of GAPDH. The results were expressed as the means ± SDs from three repeat experiments, and comparisons were evaluated by a two-tailed Student’s t test. (TIF) [file pntd.0008083.s008.tif]
